# COVID-19 Admission Rates and Changes in Care Quality in US Hospitals

**DOI:** 10.1001/jamanetworkopen.2024.13127

**Published:** 2024-05-24

**Authors:** Giacomo Meille, Pamela L. Owens, Sandra L. Decker, Thomas M. Selden, Melissa A. Miller, Jade K. Perdue-Puli, Erin N. Grace, Craig A. Umscheid, Joel W. Cohen, R. Burciaga Valdez

**Affiliations:** 1 Agency for Healthcare Research and Quality, US Department of Health and Human Services, Rockville, Maryland

## Abstract

**Importance:**

Unprecedented increases in hospital occupancy rates during COVID-19 surges in 2020 caused concern over hospital care quality for patients without COVID-19.

**Objective:**

To examine changes in hospital nonsurgical care quality for patients without COVID-19 during periods of high and low COVID-19 admissions.

**Design, Setting, and Participants:**

This cross-sectional study used data from the 2019 and 2020 Agency for Healthcare Research and Quality’s Healthcare Cost and Utilization Project State Inpatient Databases. Data were obtained for all nonfederal, acute care hospitals in 36 states with admissions in 2019 and 2020, and patients without a diagnosis of COVID-19 or pneumonia who were at risk for selected quality indicators were included. The data analysis was performed between January 1, 2023, and March 15, 2024.

**Exposure:**

Each hospital and week in 2020 was categorized based on the number of COVID-19 admissions per 100 beds: less than 1.0, 1.0 to 4.9, 5.0 to 9.9, 10.0 to 14.9, and 15.0 or greater.

**Main Outcomes and Measures:**

The main outcomes were rates of adverse outcomes for selected quality indicators, including pressure ulcers and in-hospital mortality for acute myocardial infarction, heart failure, acute stroke, gastrointestinal hemorrhage, hip fracture, and percutaneous coronary intervention. Changes in 2020 compared with 2019 were calculated for each level of the weekly COVID-19 admission rate, adjusting for case-mix and hospital-month fixed effects. Changes during weeks with high COVID-19 admissions (≥15 per 100 beds) were compared with changes during weeks with low COVID-19 admissions (<1 per 100 beds).

**Results:**

The analysis included 19 111 629 discharges (50.3% female; mean [SD] age, 63.0 [18.0] years) from 3283 hospitals in 36 states. In weeks 18 to 48 of 2020, 35 851 hospital-weeks (36.7%) had low COVID-19 admission rates, and 8094 (8.3%) had high rates. Quality indicators for patients without COVID-19 significantly worsened in 2020 during weeks with high vs low COVID-19 admissions. Pressure ulcer rates increased by 0.09 per 1000 admissions (95% CI, 0.01-0.17 per 1000 admissions; relative change, 24.3%), heart failure mortality increased by 0.40 per 100 admissions (95% CI, 0.18-0.63 per 100 admissions; relative change, 21.1%), hip fracture mortality increased by 0.40 per 100 admissions (95% CI, 0.04-0.77 per 100 admissions; relative change, 29.4%), and a weighted mean of mortality for the selected indicators increased by 0.30 per 100 admissions (95% CI, 0.14-0.45 per 100 admissions; relative change, 10.6%).

**Conclusions and Relevance:**

In this cross-sectional study, COVID-19 surges were associated with declines in hospital quality, highlighting the importance of identifying and implementing strategies to maintain care quality during periods of high hospital use.

## Key Points

**Question:**

What was the association of COVID-19 admission rates with changes in hospital care quality for patients without COVID-19 in 2020?

**Findings:**

In this cross-sectional study of 3283 acute care hospitals in 36 states and more than 19 million patient discharges, pressure ulcers and in-hospital mortality for nonsurgical care increased in 2020 during weeks with high COVID-19 admissions compared with weeks with low COVID-19 admissions. Increases were statistically significant and clinically meaningful; for example, pressure ulcer, heart failure mortality, and hip fracture mortality rates all increased by at least 20% during weeks with high compared with low COVID-19 admissions.

**Meaning:**

These findings suggest that COVID-19 surges were associated with decreases in hospital quality, highlighting the importance of future strategies to maintain care quality during periods of high use.

## Teaser

This cross-sectional study examines changes in hospital nonsurgical care quality for patients without COVID-19 during periods of high and low COVID-19 admissions in the US.

## Introduction

The COVID-19 pandemic posed numerous challenges to maintaining care quality in US hospitals.^1^ During surges, hospitals experienced unprecedented strain, with increased volumes of patients with severe illness and shortages of staff, beds, and supplies.^1,2,3^ However, hospitals often experienced few or no COVID-19 admissions, and during these periods occupancy levels were lower than in 2019.^4^ Tracking how patient outcomes changed in response to COVID-19 admissions and occupancy fluctuations may offer insight into the association between hospital strain and care quality.

Previous studies have shown that the strain from COVID-19 surges was associated with increased in-hospital mortality.^5,6,7^ However, interpreting this finding is complicated by changes in patient case mix during surges, leading studies to differentiate between patients with COVID-19 and patients without COVID-19. An extensive literature has found that hospital strain was associated with increases in mortality for patients with COVID-19,^8,9,10,11,12,13,14,15,16^ and a smaller set of studies has found increases in mortality among patients without COVID-19.^5,7,17^ Studies that examined patients without COVID-19 relied on Medicare claims,^17^ a convenience sample of US insurance claims,^5^ or United Kingdom data.^7^ None of these studies examined a representative sample of hospitalizations of US patients or changes in patient case mix during surges. Related studies that examined patient morbidity during COVID-19 surges focused exclusively on health care–associated infections.^18,19,20,21,22^

This study investigated the association between COVID-19 admission rates and hospital care quality for patients without COVID-19 using the Agency for Healthcare Research and Quality’s (AHRQ’s) Quality Indicators (QIs). The QIs are designed to differentiate care quality among hospitals or within hospitals over time with minimal case-mix bias^23^ and are widely used to inform quality improvement and pay-for-performance initiatives.^23,24,25^ We first examined changes in the case mix compared with 2019 as a function of COVID-19 admissions. We then examined the case-mix–adjusted QI rates for pressure ulcers, a nursing-sensitive complication, and for in-hospital mortality related to nonsurgical care for selected conditions.

## Methods

This cross-sectional study was reviewed by AHRQ’s human protection administrator, who determined that the project did not constitute human participants research and did not require additional review by an institutional review board or informed consent. The study followed the Strengthening the Reporting of Observational Studies in Epidemiology (STROBE) reporting guideline.

### Data

We used the AHRQ Healthcare Cost and Utilization Project State Inpatient Databases for 36 states (eTable 1 in Supplement 1). These data cover all discharges from all nonfederal acute care hospitals in the included states. Hospitals were included if they admitted patients in 2019 and 2020, resulting in 3283 hospitals (76.1% of US nonfederal acute care hospitals).^26^ The data covered 2019, the 2020 prepandemic period (weeks 1-10), and the 2020 pandemic period (weeks 18-48). We excluded weeks 11 to 17, weeks 49 to 52, and patients with COVID-19 or pneumonia. Admissions from weeks 11 to 17 were excluded because of COVID-19 testing shortages early in the pandemic,^27,28^ which may have led to difficulty identifying patients without COVID-19. Admissions during weeks 49 to 52 were excluded because patients discharged in 2021 were not included in the 2020 Healthcare Cost and Utilization Project State Inpatient Databases. Patients with pneumonia were excluded because they may have had undiagnosed COVID-19, especially early in the pandemic due to variable reliability, availability, and use of testing. The eFigure in Supplement 1 shows that a high proportion of pneumonia admissions throughout 2020 included a COVID-19 diagnosis.

The State Inpatient Databases consist of detailed discharge-level information, including patient disposition, clinical condition codes, demographics, admission date, and hospital identifiers. In sample statistics, we report the distribution of patient race and ethnicity for the 3 most common categories (Hispanic, non-Hispanic Black, non-Hispanic White) and other race and ethnicity. Discharge records on race and ethnicity are ideally based on patient self-reporting but may be collected by hospital staff based on observation.^29,30^

### Outcomes

Our primary outcome of interest was the quality of hospital care, as measured by AHRQ QIs for patient safety (Patient Safety Indicators [PSIs]) and inpatient quality (Inpatient Quality Indicators [IQIs]).^31^ We excluded pediatric, obstetric, and surgical QIs, as these relate to distinct patient populations and hospital units that were subject to different policies (eg, moratoriums on elective procedures) and changes in case mix. We further limited QIs based on a power analysis, selecting indicators with sufficient observations to detect a 15% change at the standard 5% significance level (details provided in eAppendix 1 in Supplement 1). This power analysis was conducted before the main analyses and aimed to reduce the probability of type I and type II errors (ie, false-positive and false-negative results).

The power analysis yielded 7 indicators from the broader set of nonsurgical, nonobstetric, nonpediatric QIs (eTable 2 in Supplement 1). The selected QIs were the PSI for pressure ulcer rate (PSI 03); IQIs measuring in-hospital mortality for 5 medical conditions (IQI 15, acute myocardial infarction mortality; IQI 16, heart failure mortality; IQI 17, acute stroke mortality; IQI 18, gastrointestinal hemorrhage mortality; and IQI 19, hip fracture mortality); and in-hospital mortality associated with a nonsurgical invasive procedure for coronary artery disease (IQI 30, percutaneous coronary intervention mortality). Detailed definitions for these QIs are provided in eTable 3 in Supplement 1. Prior literature has shown that many of these indicators are associated with nurse staffing,^32,33,34^ and all these indicators vary across hospitals and are associated with poorer care quality.^23,31,35^

Each QI specifies a denominator that corresponds to the patients at risk and a numerator for the adverse outcome (pressure ulcer or in-hospital death). Our first set of outcomes examined patient case mix, which may confound analyses of care quality. Changes in case mix may have increased or decreased patient severity, depending on factors such as risk aversion for COVID-19 infection, time sensitivity of conditions, and hospital and public health policies regarding elective admissions. For each QI, we examined the number of patients at risk (patient volume) and their mean age and mortality-weighted mean Elixhauser Comorbidity Index Refined for *International Classification of Diseases, Tenth Revision, Clinical Modification*^36^ (hereafter, comorbidity index). The comorbidity index is a composite score that weights 38 comorbidities based on their correlation with in-hospital mortality. A robustness check was conducted using the readmission-weighted comorbidity index, which weights the comorbidities based on their correlation with readmissions.^36^ (The number of comorbidities assigned a nonzero weight is 33 and 30 for the mortality and readmission comorbidity indices, respectively; additional details on methodology are provided in eAppendix 2, and weights are provided in eTable 4 in Supplement 1.)

Our primary outcomes of interest were the rates of adverse outcomes for each QI. We used discharge-level indicator variables for pressure ulcer or in-hospital death within the sample of patients at risk for each outcome.

### Exposure Variable

The exposure variable was the rate of COVID-19 admissions per 100 hospital beds measured at the hospital-week level. In previous work, our group showed that COVID-19 admissions were associated with fluctuations in occupancy.^4^ Specifically, the previous study estimated that hospital-weeks in 2020 with low COVID-19 admission rates were associated with a 12.7% reduction in inpatient occupancy and a 5.4% reduction in intensive care unit (ICU) occupancy compared with the same hospital-weeks in 2019. In contrast, hospital-weeks with high COVID-19 admission rates were associated with a 7.9% increase in inpatient occupancy and a 67.8% increase in ICU occupancy. Our group’s prior study also provides detail on the distribution of COVID-19 admission rates over time, states, and types of hospitals.^4^

COVID-19 admissions were identified using *International Classification of Diseases, Tenth Revision, Clinical Modification* codes U07.1 and J12.82 for patients aged 1 year or older. Following the prior study,^4^ we used 5 indicators for the weekly COVID-19 admission rate per 100 hospital beds to allow a nonlinear relationship with outcomes: less than 1 (low), 1 to 4.9, 5 to 9.9, 10 to 14.9, and 15 or greater (high).

### Statistical Analysis

Data were analyzed between January 1, 2023, and March 15, 2024. For our case-mix analyses, we compared patient volume, mean age, and mean comorbidities for each hospital-week in 2020 with the corresponding hospital-week in 2019. Each outcome was regressed on the categorical COVID-19 admission rate interacted with indicators for the 2020 prepandemic and 2020 pandemic periods (2019 was the omitted baseline period). In addition, we controlled for hospital-week indicators. Regressions for age and comorbidities were weighted by patient volume in each hospital-week. For difference-in-differences analyses, the econometrics literature recommends clustering standard errors at the level of treatment to account for correlations in outcomes over time.^37,38^ As the COVID-19 admission rate is a hospital-week–level variable, we followed our prior study and clustered standard errors at the hospital level in all regressions.^4^

For pressure ulcer and mortality QIs, we estimated discharge-level regressions that compared each hospital-month in 2020 with the corresponding hospital-month in 2019. Thus, discharges during hospital-weeks with high and low COVID-19 admissions were compared with discharges during the corresponding hospital-month in 2019. Each QI was regressed on the categorical hospital-week–level COVID-19 admission rate interacted with the period (2020 prepandemic or 2020 pandemic), controlling for confounders by including hospital-month indicators and case-mix controls. Case-mix controls included an age spline interacted with patient sex, indicators for Medicare Severity Diagnosis Related Groups (or major diagnostic categories when Medicare Severity Diagnosis Related Groups had <1000 observations), and comorbidities. We included separate indicators for each of the 38 comorbidities underlying the comorbidity index,^36^ thereby allowing associations between comorbidities and QIs to be determined empirically rather than using the predetermined weights in the index.

The tables in the body of the article report the estimated associations during the pandemic period. They separately report changes in case mix and QIs during hospital-weeks with low COVID-19 admissions, changes during high COVID-19 admissions, and the difference. The difference corresponds to the incremental change during high COVID-19 admissions compared with the change during low COVID-19 admissions. In addition to reporting changes for each QI, we report a summary measure for the selected mortality QIs. This measure is the sum of coefficients for patient volume and the patient volume–weighted mean of coefficients for age, the comorbidity index, and the mortality QIs. The summary measures reduced the probability of false positives from multiple hypothesis testing and increased statistical power by pooling across multiple QIs, which increased the sample size.

Unless otherwise noted, results discussed are statistically significant at the 5% level (2-sided). Analyses were performed using Stata/MP, version 18 software (StataCorp LLC).

## Results

Our sample consisted of 19 111 629 discharges from 3283 hospitals during 2019 and 2020. Table 1 shows that during the 2020 pandemic period, 35 851 hospital-weeks (36.7%) had low COVID-19 admission rates, and 8094 (8.3%) had high rates. The mean (SD) age of patients was 63.0 (18.0) years and 50.3% were female (compared with 49.7% male). The distribution of patient race and ethnicity was 9.9% Hispanic, 16.6% non-Hispanic Black; 66.4% non-Hispanic White; and 5.3% other race or ethnicity (including non-Hispanic Asian and non-Hispanic multiracial or other), with 1.8% missing race or ethnicity information. Additional sample characteristics, including characteristics for patients at risk of each QI, are provided in eTable 6 in Supplement 1.

**Table 1.** Distribution of Hospital-Weeks and Admissions by COVID-19 Admissions During Weeks 18 to 48 of 2020^a^

Admission rate per 100 beds	No. (%)	
	Hospital-wk (n = 97 718)	Admissions (n = 8 342 237)
<1.0	35 851 (36.7)	3 125 598 (37.5)
1.0-4.9	29 843 (30.5)	2 848 497 (34.1)
5.0-9.9	16 640 (17.0)	1 417 380 (17.0)
10.0-14.9	7290 (7.5)	534 491 (6.4)
≥15.0	8094 (8.3)	416 271 (5.0)

^a^ Source: Agency for Healthcare Research and Quality, Healthcare Cost and Utilization Project State Inpatient Databases, 36 States, 2019-2020.

Table 2 presents changes in the number of patients without COVID-19 at risk for each QI. For each measure, compared with 2019, patient volume decreased in 2020, with larger decreases during weeks with high COVID-19 admissions. During weeks with low COVID-19 admissions, the number of patients at risk for pressure ulcers decreased by 2.69 per hospital-week (95% CI, 2.45-2.92 per hospital-week), a 4.0% decrease compared with the mean (SE) in 2019 of 66.98 (1.64) per hospital-week. The number of patients at risk of any selected in-hospital mortality QI decreased by 0.55 per hospital-week (95% CI, 0.49-0.62 per hospital-week), a 3.3% decrease relative to the 2019 mean (SE) of 16.69 (0.41) per hospital-week. During weeks with high COVID-19 admissions, the number of patients at risk for pressure ulcers decreased by 14.75 per hospital-week (95% CI, 13.60-15.89 per hospital-week; relative change, 22.0%), and the number of patients at risk for any selected in-hospital mortality QI decreased by 3.48 per hospital-week (95% CI, 3.17-3.79 per hospital-week; relative change, 20.9%). For each QI, the decrease in patient volume was larger during weeks with high COVID-19 admissions relative to weeks with low COVID-19 admissions.

**Table 2.** Volume of At-Risk Patients for Selected Quality Indicators in 2019 and Change in 2020 During Weeks of Low and High COVID-19 Admissions^a^

Quality Indicator	No. of hospital-wk	No. of patients at risk per hospital-wk			
		2019, Mean (SE)^b^	Estimate (95% CI)^b^^,^^c^		
			Change during low COVID-19 wk	Change during high COVID-19 wk	Difference in the change during high vs low COVID-19 wk
PSI 03: pressure ulcer	254 770	66.98 (1.64)	−2.69 (−2.92 to −2.45)	−14.75 (−15.89 to −13.60)	−12.06 (−13.20 to −10.92)
Sum of mortality indicators below	1 528 620	16.69 (0.41)	−0.55 (−0.62 to −0.49)	−3.48 (−3.79 to −3.17)	−2.93 (−3.24 to −2.62)
IQI 15: AMI mortality	254 770	2.72 (0.08)	−0.16 (−0.18 to −0.13)	−0.60 (−0.68 to −0.52)	−0.44 (−0.52 to −0.36)
IQI 16: heart failure mortality	254 770	5.12 (0.12)	−0.20 (−0.23 to −0.18)	−1.44 (−1.56 to −1.31)	−1.23 (−1.35 to −1.11)
IQI 17: acute stroke mortality	254 770	2.93 (0.08)	−0.06 (−0.08 to −0.05)	−0.43 (−0.50 to −0.36)	−0.37 (−0.44 to −0.29)
IQI 18: GI hemorrhage mortality	254 770	2.52 (0.06)	−0.06 (−0.07 to −0.04)	−0.42 (−0.49 to −0.36)	−0.37 (−0.43 to −0.30)
IQI 19: hip fracture mortality	254 770	1.29 (0.03)	−0.01 (−0.02 to 0.00)	−0.15 (−0.19 to −0.11)	−0.15 (−0.19 to −0.10)
IQI 30: PCI mortality	254 770	2.11 (0.06)	−0.06 (−0.08 to −0.04)	−0.44 (−0.50 to −0.38)	−0.38 (−0.44 to −0.31)

Abbreviations: AMI, acute myocardial infarction; GI, gastrointestinal; IQI, Inpatient Quality Indicator; PCI, percutaneous coronary intervention; PSI, Patient Safety Indicator.

^a^ Source: Agency for Healthcare Research and Quality, Healthcare Cost and Utilization Project State Inpatient Databases, 36 States, 2019-2020.

^b^ Standard errors were clustered at the hospital level.

^c^ Changes were reported for weeks 18 to 48 based on a hospital-week–level linear regression of 2019 and 2020 data. Fixed effects for hospital-weeks are included in each regression.

Table 3 presents the results for the mean age of patients at risk for each QI. Relative to 2019, the mean patient age decreased for each QI in both weeks with low and weeks with high COVID-19 admissions. During weeks with low COVID-19 admissions, patient age decreased by 0.47 years (95% CI, 0.39-0.55 years) for patients at risk of pressure ulcers, a 0.7% decrease, and by 0.58 years (95% CI, 0.47-0.69 years) for patients at risk for any selected mortality QI, a 0.8% decrease. During weeks with high COVID-19 admissions, patient age decreased by 0.76 years (95% CI, 0.66-0.86 years; relative change, 1.1%) for patients at risk for pressure ulcers and by 0.73 years (95% CI, 0.60-0.86 years; relative change, 1.1%) for patients at risk of any selected mortality QI. Compared with weeks with low COVID-19 admissions, the decrease in age was larger for all QIs during weeks with high COVID-19 admissions. However, the incremental decrease was only statistically significant for patients at risk of pressure ulcers.

**Table 3.** Mean Age of At-Risk Patients for Selected Quality Indicators in 2019 and Change in 2020 During Weeks of Low and High COVID-19 Admissions^a^

Quality Indicator	No. of hospital-wk	Mean age of at-risk patients at hospital-wk level, y			
		2019, Mean (SE)^b^^,^^c^	Estimate (95% CI)^b^^,^^c^^,^^d^		
			Change during low COVID-19 wk	Change during high COVID-19 wk	Difference in the change during high vs low COVID-19 wk
PSI 03: pressure ulcer	235 268	62.82 (0.13)	−0.47 (−0.55 to −0.39)	−0.76 (−0.86 to −0.66)	−0.29 (−0.42 to −0.16)
Mean of mortality indicators below^b^	667 592	69.46 (0.09)	−0.58 (−0.69 to −0.47)	−0.73 (−0.86 to −0.60)	−0.15 (−0.32 to 0.02)
IQI 15: AMI mortality	102 014	66.31 (0.07)	−0.32 (−0.55 to −0.08)	−0.53 (−0.82 to −0.24)	−0.22 (−0.58 to 0.15)
IQI 16: heart failure mortality	149 790	70.75 (0.14)	−1.23 (−1.42 to −1.04)	−1.37 (−1.58 to −1.15)	−0.14 (−0.42 to 0.15)
IQI 17: acute stroke mortality	111 338	69.26 (0.10)	−0.54 (−0.79 to −0.29)	−0.49 (−0.78 to −0.21)	0.05 (−0.34 to 0.43)
IQI 18: GI hemorrhage mortality	119 286	67.49 (0.11)	−0.38 (−0.69 to −0.06)	−0.60 (−0.96 to −0.23)	−0.22 (−0.69 to 0.25)
IQI 19: hip fracture mortality	93 292	81.96 (0.04)	−0.03 (−0.24 to 0.18)	−0.27 (−0.54 to −0.01)	−0.24 (−0.57 to 0.09)
IQI 30: PCI mortality	91 872	66.17 (0.07)	0.05 (−0.18 to 0.28)	−0.22 (−0.52 to 0.09)	−0.26 (−0.64 to 0.12)

Abbreviations: AMI, acute myocardial infarction; GI, gastrointestinal; IQI, Inpatient Quality Indicator; PCI, percutaneous coronary intervention; PSI, Patient Safety Indicator.

^a^ Source: Agency for Healthcare Research and Quality, Healthcare Cost and Utilization Project State Inpatient Databases, 36 States, 2019-2020.

^b^ Means, regressions, and mean of mortality indicators were weighted by the number of admissions for each quality indicator in each hospital-week.

^c^ Standard errors were clustered at the hospital level.

^d^ Changes were reported for weeks 18 to 48 based on a hospital-week–level linear regression of 2019 and 2020 data. Fixed effects for hospital and weeks were included in each regression.

Table 4 presents the comorbidity index for patients at risk for each QI. For 5 of 7 QIs, the estimated change during weeks with low COVID-19 admissions was positive, indicating that patients at risk had more comorbidities compared with 2019. For 3 of 7 QIs, in weeks with high COVID-19 admissions, patients at risk also had more comorbidities compared with 2019. For all QIs, the number of comorbidities decreased among patients at risk admitted during weeks with high compared with low COVID-19 admissions. This difference was statistically significant for pressure ulcers (−0.18; 95% CI, −0.29 to −0.07), heart failure mortality (−0.29; 95% CI, −0.53 to −0.04), PCI mortality (−0.56; 95% CI, −0.96 to −0.16), and the weighted mean of the mortality QIs (−0.33; 95% CI, −0.50 to −0.16). Analyses that used the readmission-weighted index showed similar trends (eTable 5 in Supplement 1).

**Table 4.** Elixhauser Comorbidity Index for In-Hospital Mortality Among At-Risk Patients for Selected Quality Indicators in 2019 and Change in 2020 During Weeks of Low and High COVID-19 Admissions^a^

Quality Indicator	No. of hospital-wk	Mean Elixhauser Comorbidity Index Refined for in-hospital mortality among at-risk patients at the hospital-wk level			
		2019, Mean (SE)^b^^,^^c^	Estimate (95% CI)^b^^,^^c^^,^^d^		
			Change during low COVID-19 wk	Change during high COVID-19 wk	Difference in the change during high vs low COVID-19 wk
PSI 03: pressure ulcer	235 268	7.12 (0.07)	0.51 (0.44 to 0.58)	0.33 (0.24 to 0.41)	−0.18 (−0.29 to −0.07)
Mean of mortality indicators below^b^	667 592	10.76 (0.06)	0.23 (0.11 to 0.34)	−0.11 (−0.24 to 0.02)	−0.33 (−0.50 to −0.16)
IQI 15: AMI mortality	102 014	6.31 (0.06)	−0.18 (−0.42 to 0.07)	−0.48 (−0.77 to −0.19)	−0.30 (−0.67 to 0.06)
IQI 16: heart failure mortality	149 790	16.54 (0.05)	−0.22 (−0.39 to −0.06)	−0.51 (−0.69 to −0.32)	−0.29 (−0.53 to −0.04)
IQI 17: acute stroke mortality	111 338	11.86 (0.11)	0.61 (0.33 to 0.90)	0.40 (0.07 to 0.73)	−0.21 (−0.65 to 0.23)
IQI 18: GI hemorrhage mortality	119 286	9.57 (0.07)	0.93 (0.63 to 1.23)	0.55 (0.21 to 0.90)	−0.38 (−0.83 to 0.07)
IQI 19: hip fracture mortality	93 292	6.32 (0.06)	0.37 (0.07 to 0.67)	−0.03 (−0.43 to 0.37)	−0.41 (−0.90 to 0.09)
IQI 30: PCI mortality	91 872	4.71 (0.06)	0.37 (0.09 to 0.64)	−0.20 (−0.49 to 0.09)	−0.56 (−0.96 to −0.16)

Abbreviations: AMI, acute myocardial infarction; GI, gastrointestinal; IQI, Inpatient Quality Indicator; PCI, percutaneous coronary intervention; PSI, Patient Safety Indicator.

^a^ Source: Agency for Healthcare Research and Quality, Healthcare Cost and Utilization Project State Inpatient Databases, 36 States, 2019-2020.

^b^ Means, regressions, and mean of mortality indicators were weighted by the number of admissions for each quality indicator in each hospital-week.

^c^ Standard errors were clustered at the hospital level.

^d^ Changes were reported for weeks 18 to 48 based on a hospital-week-level linear regression of 2019 and 2020 data. Fixed effects for hospital and weeks are included in each regression.

Table 5 presents QI changes during weeks with low and high COVID-19 admissions, adjusted for age, sex, comorbidities, and hospital-month fixed effects. Compared with 2019, during weeks with low COVID-19 admissions, pressure ulcers decreased by 0.12 per 1000 admissions (95% CI, 0.06-0.18 per 1000 admissions; relative change, 32.4%), and acute stroke mortality decreased by 0.54 per 100 admissions (95% CI, 0.22-0.86 per 100 admissions; relative change, 10.1%). Changes for other QIs were not statistically significant. In contrast, compared with 2019, during weeks with high COVID-19 admissions, in-hospital mortality increased for heart failure (0.42 per 100 admissions; 95% CI, 0.24-0.59 per 100 admissions; relative change, 22.1%), as well as for gastrointestinal hemorrhage (0.29 per 100 admissions; 95% CI, 0.06-0.52 per 100 admissions; relative change, 16.3%) and hip fracture (0.33 per 100 admissions; 95% CI, 0.05-0.61 per 100 admissions; relative change, 24.2%) (eTable 7 in Supplement 1). The mean change in in-hospital mortality across the mortality QIs (calculated by the summary measure) was an increase of 0.22 per 100 admissions (95% CI, 0.10-0.34 per 100 admissions; relative change, 7.8%) during weeks with high COVID-19 admissions. The change in pressure ulcer rate was not statistically significant during those weeks. For all QIs, adverse outcomes increased during weeks with high compared with low COVID-19 admissions, with statistically significant increases for pressure ulcers of 0.09 per 1000 admissions (95% CI, 0.01-0.17 per 1000 admissions; relative change, 24.3%), in-hospital mortality for heart failure of 0.40 per 100 admissions (95% CI, 0.18-0.63 per 100 admissions; relative change, 21.1%), hip fracture of 0.40 per 100 admissions (95% CI, 0.04-0.77 per 100 admissions; relative change, 29.4%), and the weighted mean for the mortality QIs of 0.30 per 100 admissions (95% CI, 0.14-0.45 per 100 admissions; relative change, 10.6%).

**Table 5.** Selected Quality Indicators in 2019 and Changes in 2020 During Weeks of Low and High COVID-19 Admissions^a^

Quality Indicator	No. of discharges	Quality Indicator means, pressure ulcers per 1000 at-risk admissions, or in-hospital mortality per 100 at-risk admissions				
		2019, Mean (SE)^b^^,^^c^	Estimate (95% CI)^c^^,^^d^			
			Change during low COVID-19 wk	Change during high COVID-19 wk	Difference in the change during high vs low COVID-19 wk
PSI 03: pressure ulcer	17 779 128	0.37 (0.02)	−0.12 (−0.18 to −0.06)	−0.03 (−0.10 to 0.03)	0.09 (0.01 to 0.17)
Mean of mortality indicators listed^e^	4 429 736	2.83 (0.03)	−0.08 (−0.17 to 0.02)	0.22 (0.10 to 0.34)	0.30 (0.14 to 0.45)
IQI 15: AMI mortality	703 457	3.87 (0.04)	0.02 (−0.16 to 0.20)	0.21 (−0.04 to 0.45)	0.19 (−0.12 to 0.50)
IQI 16: heart failure mortality	1 354 741	1.90 (0.03)	0.02 (−0.13 to 0.16)	0.42 (0.24 to 0.59)	0.40 (0.18 to 0.63)
IQI 17: acute stroke mortality	787 982	5.36 (0.10)	−0.54 (−0.86 to −0.22)	−0.17 (−0.50 to 0.17)	0.38 (−0.09 to 0.84)
IQI 18: GI hemorrhage mortality	675 849	1.78 (0.03)	0.09 (−0.12 to 0.29)	0.29 (0.06 to 0.52)	0.20 (−0.10 to 0.51)
IQI 19: hip fracture mortality	348 496	1.36 (0.03)	−0.07 (0.30 to 0.16)	0.33 (0.05 to 0.61)	0.40 (0.04 to 0.77)
IQI 30: PCI mortality	559 211	2.41 (0.04)	0.03 (−0.21 to 0.27)	0.14 (−0.19 to 0.46)	0.11 (−0.29 to 0.51)

Abbreviations: AMI, acute myocardial infarction; GI, gastrointestinal; IQI, Inpatient Quality Indicator; PCI, percutaneous coronary intervention; PSI, Patient Safety Indicator.

^a^ Source: Agency for Healthcare Research and Quality, Healthcare Cost and Utilization Project State Inpatient Databases, 36 States, 2019-2020.

^b^ Means in 2019 calculated at the discharge level.

^c^ Standard errors clustered at the hospital level.

^d^ Changes were reported for weeks 18 to 48 based on a discharge-level linear regression of 2019 and 2020 data. Each regression controlled for sex, an age spline, interactions between sex and the age spline, Medicare Severity Diagnosis Related Groups (or major diagnostic category if <1000 observations for a Medicare Severity Diagnosis Related Group), and 38 comorbidities. Fixed effects for hospital-months are included in each regression.

^e^ Mean was weighted by number of discharges for each mortality Quality Indicator.

In eTable 7 in Supplement 1, we show the full regression results, including estimates for all levels of COVID-19 admissions. The results suggest that the rate of most QIs increased as the COVID-19 admission rate increased.

## Discussion

This cross-sectional study examined selected QIs for patients without COVID-19, using 2019 to 2020 data from all nonfederal acute care hospitals in 36 states. We compared changes in QIs in 2020 with 2019 during weeks with high and low COVID-19 admission rates. After adjusting for case-mix and hospital-month fixed effects, we found increased rates of pressure ulcers and in-hospital mortality in weeks with high compared with low COVID-19 admissions, suggesting that health care quality decreased during surges.

In contrast to previous studies,^5,7,17^ we used all-payer discharges from all nonfederal acute care hospitals in 36 states and examined narrowly defined QIs that may be more sensitive to differences in care quality and staffing shortages than all-cause mortality. Furthermore, we adopted stricter exclusion criteria to minimize observations with undiagnosed COVID-19 by dropping admissions during the early pandemic (when hospitals experienced shortages of laboratory testing and supplies) and admissions with pneumonia (which may have been undiagnosed COVID-19). We also improved upon previous studies by comparing outcomes in each hospital-month during 2020 with the same hospital-month in 2019 to avoid confounding COVID-19 surge effects within hospitals with effects due to changes in volumes across hospitals. Finally, we contribute to the literature on morbidity during the pandemic. Previous studies were limited to health care–associated infections,^18,19,20,21^ and analyses of patients without COVID-19 did not compare infection rates by level of COVID-19 burden.^22^

To our knowledge, our study is the first to estimate changes in patient case mix during periods with low and high COVID-19 admission rates. As noted by others, several factors may have affected case mix during the pandemic, including federal policies restricting elective admissions, hospitals conserving resources to treat patients with COVID-19, and individuals avoiding settings of care in which they may become infected with COVID-19.^39,40,41^ Our results show decreases in age and comorbidities in weeks with high vs low COVID-19 admissions. They suggest that, on average, patients admitted during weeks with high COVID-19 admissions were healthier prior to admission compared with weeks with low COVID-19 admissions.

Our findings show that compared with 2019, pressure ulcer rates decreased 32.4% during weeks with low COVID-19 admissions in 2020, whereas we found no evidence of a change during weeks with high COVID-19 admissions in 2020. The significant difference between periods of high and low COVID-19 admissions reflects a positive association between COVID-19 burden and pressure ulcer rates, a key measure of complications from care. Our group’s previous study of occupancy rates during the pandemic offers essential context for these results, finding that occupancy declined slightly during weeks with low COVID-19 admissions and increased substantially during weeks with high COVID-19 admissions, especially for ICUs.^4^ The decline in the rate of pressure ulcers during weeks with low COVID-19 admissions may be related to an increased nurse-to-patient ratio during periods with decreased patient volume or an increased emphasis and training on preventing pressure ulcers during the pandemic.^42,43^ However, when hospitals are overburdened with severely ill patients, they may lack the necessary resources or staff to prevent pressure ulcers, a complication of hospital care particularly sensitive to nurse staffing.^32^

The results for in-hospital mortality followed a different pattern. For heart failure, hip fracture, and our summary measure of in-hospital mortality for nonsurgical care, we did not find evidence of a change in mortality during weeks in 2020 with low COVID-19 admissions. However, we found increases in mortality during weeks in 2020 with high COVID-19 admissions. Several factors may have contributed to these decreases in care quality and worse outcomes, including staff shortages, assignment of inadequately trained staff to medical floors for patients without COVID-19, restrictive visitor policies limiting family support at the bedside, inability to monitor and manage changes in patient disposition, inadequate or lack of protective equipment to prevent the spread of infections due to supply chain limitations, and impaired quality improvement processes.^1,2,44,45,46^ Further research is needed to explore these complex factors and identify practices that improve health care resiliency. Strategies such as building an organizational culture that prioritizes patient and workforce safety and maintaining nurse to patient ratios may help to build resiliency during periods of strain.^47,48,49^

### Limitations

Our study has some limitations. First, although our models included extensive case-mix controls, our results may reflect spurious correlations between unmeasured dimensions of patient severity and fluctuations in COVID-19 admissions. For example, comorbidities may be imperfectly captured by administrative data collected primarily for reimbursement purposes.^50,51^ Notwithstanding these limitations, our case-mix analyses found fewer comorbidities and younger age during weeks with high vs low COVID-19 admissions. If other unmeasured dimensions of patient severity also decreased during weeks with high vs low COVID-19 admissions, our results may have underestimated the associations of COVID-19 surges with care quality.

Second, we were unable to control for subnational trends, including supply and staff shortages. These factors may have covaried with COVID-19 admission rates and affected QIs. Our hospital-month fixed effects controlled for fixed hospital characteristics, and by comparing changes during periods with high and low COVID-19 admissions, we differenced out national trends.

## Conclusions

In this cross-sectional study, COVID-19 surges were associated with declines in hospital quality for patients without COVID-19. The results highlight the importance of implementing strategies to maintain health care quality during periods of high hospital use, particularly during public health emergencies such as the COVID-19 pandemic. Mitigating staffing shortages, building a safety culture and adopting related organizational best practices, and harnessing new technologies to reduce burden may improve hospital resilience during periods of high use.

## Notes

Supplement 1. **eTable 1.** Included and Excluded States **eFigure.** Percentage of 2020 Admissions With COVID-19, Among Admissions With Pneumonia **eAppendix 1.** Selection of Hospital Patient Safety and Quality Indicators **eTable 2.** Power Analysis for QIs (b.1) **eTable 3.** Description of Selected Quality Indicators (b.2.) **eAppendix 2.** Background and Additional Results for Elixhauser Comorbidity Index Refined **eTable 4.** List of Comorbidities in Elixhauser Comorbidity Software Refined, v2022.1, Observed In-Hospital Mortality Rates and Index Weights, Observed 30-Day All-Cause Readmission Rates and Index Weights (c.1) **eTable 5.** Elixhauser Comorbidity Index Refined for Readmission Among At-Risk Patients for Selected Quality Indicators in 2019 and Change in 2020 During Weeks of Low and High COVID-19 Admissions (c.2.) **eTable 6.** Sample Characteristics (d.1) **eTable 7.** Hospital Quality Indicators in 2019 and Adjusted Changes in 2020 by Level of COVID-19 Admissions (e.1)
eReferences

Supplement 2.
Data Sharing Statement
