# Supplementary material for: COVID-19 Admission Rates and Changes in Care Quality in US Hospitals
Source: JAMA Netw Open. 2024 May 24;7(5):e2413127. doi: 10.1001/jamanetworkopen.2024.13127 (PMC11127115; doi:10.1001/jamanetworkopen.2024.13127)
Supplement: Supplement 1. — eTable 1. Included and Excluded States eFigure. Percentage of 2020 Admissions With COVID-19, Among Admissions With Pneumonia eAppendix 1. Selection of Hospital Patient Safety and Quality Indicators eTable 2. Power Analysis for QIs (b.1) eTable 3. Description of Selected Quality Indicators (b.2.) eAppendix 2. Background and Additional Results for Elixhauser Comorbidity Index Refined eTable 4. List of Comorbidities in Elixhauser Comorbidity Software Refined, v2022.1, Observed In-Hospital Mortality Rates and Index Weights, Observed 30-Day All-Cause Readmission Rates and Index Weights (c.1) eTable 5. Elixhauser Comorbidity Index Refined for Readmission Among At-Risk Patients for Selected Quality Indicators in 2019 and Change in 2020 During Weeks of Low and High COVID-19 Admissions (c.2.) eTable 6. Sample Characteristics (d.1) eTable 7. Hospital Quality Indicators in 2019 and Adjusted Changes in 2020 by Level of COVID-19 Admissions (e.1) eReferences [file jamanetwopen-e2413127-s001.pdf]

Supplemental Online Content

Meille G, Owens PL, Decker SL, et al. COVID-19 admission rates and changes in care quality in US hospitals. *JAMA Netw Open*. 2024;7(5):e2413127.
doi:10.1001/jamanetworkopen.2024.13127

eTable 1. Included and Excluded States

eFigure. Percentage of 2020 Admissions With COVID-19, Among Admissions With Pneumonia

eAppendix 1. Selection of Hospital Patient Safety and Quality Indicators

eTable 3. Power Analysis for QIs

eTable 4. Description of Selected Quality Indicators

eAppendix 2. Background and Additional Results for Elixhauser Comorbidity Index Refined

eTable 5. List of Comorbidities in Elixhauser Comorbidity Software Refined, v2022.1, Observed In-Hospital Mortality Rates and Index Weights, Observed 30-Day All-Cause Readmission Rates and Index Weights

eTable 6. Elixhauser Comorbidity Index Refined for Readmission Among At-Risk Patients for Selected Quality Indicators in 2019 and Change in 2020 During Weeks of Low and High COVID-19 Admissions

eTable 7. Sample Characteristics

eTable 8. Hospital Quality Indicators in 2019 and Adjusted Changes in 2020 by Level of COVID-19 Admissions

eReferences

This supplemental material has been provided by the authors to give readers additional information about their work.

© 2024 Meille G et al. *JAMA Network Open.*

eTable 1. Included and Excluded States[a]

States excluded from the analysis due to data limitations (12 states)	
Not in HCUP (2 states)	AL ID
Did not provide admission date in HCUP (4 states)	CO CT PA WA
Did not include data element to calculate the PSIs or IQIs (6 states)	DE OK NH SD VT WI WY
Data not available at the beginning of the study (2 states)	NE NY
States included in the analysis (36 including DC)	AK AZ AR CA DC FL GA HI IL IN IA KS KY LA ME MD MA MI MN MS MO MT NV NJ NM NC ND OH OR RI SC TN TX UT VA WV

Abbreviations: AHRQ, Agency for Healthcare Research and Quality; HCUP, Healthcare Cost and Utilization Project; PSI, patient safety indicator; IQI, inpatient quality indicator.
[a]Source: AHRQ HCUP State Inpatient Databases (SID), 2019-2020.

© 2024 Meille G et al. *JAMA Network Open.*

eFigure. Percentage of 2020 Admissions with COVID-19, Among Admissions with Pneumonia[a]

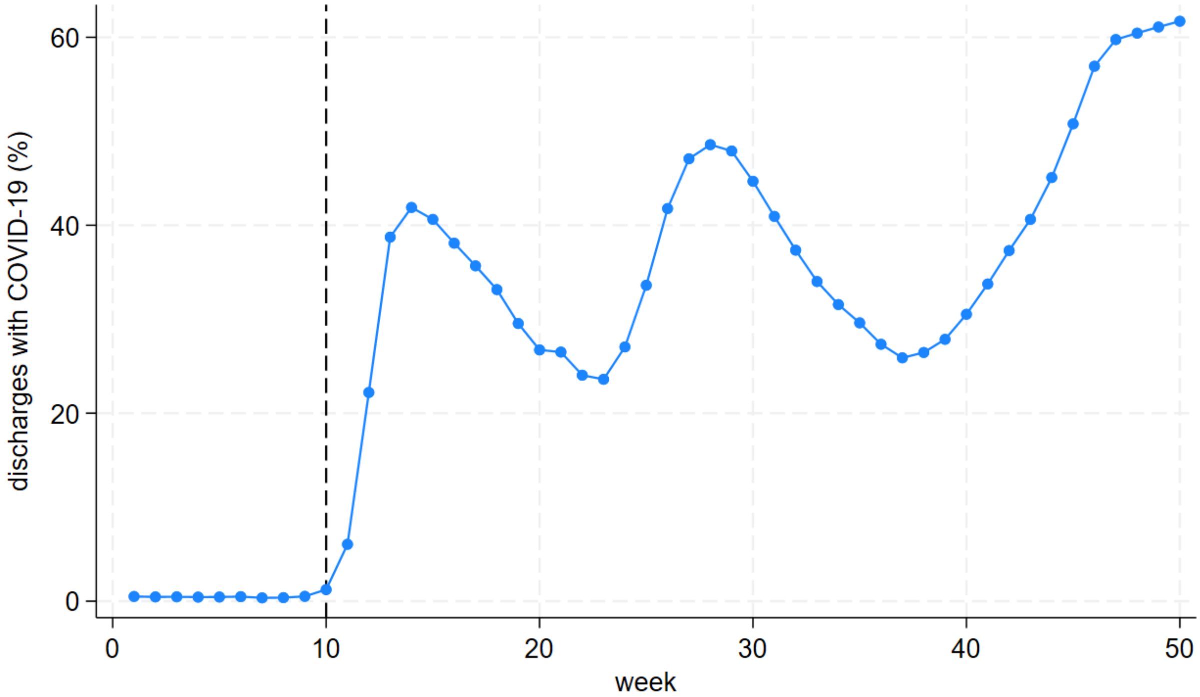

[a]Source: Agency for Healthcare Research and Quality (AHRQ), Healthcare Cost and Utilization Project (HCUP) State Inpatient Databases (SID), 36 states, 2020

© 2024 Meille G et al. *JAMA Network Open.*

eAppendix 1. Selection of Hospital Patient Safety and Quality Indicators

We examined measures of adult non-surgical, non-obstetric care using the Agency for Healthcare Research and Quality (AHRQ) Quality Indicators (QIs) v2022.01, including the Inpatient Quality Indicators (IQIs) and Patient Safety Indicators (PSIs).[1,2] The AHRQ QIs are created to assess quality differences across hospitals and assist hospitals in identifying areas in need of quality improvement.[3] Although the AHRQ QIs are no longer submitted to the National Quality Forum for national endorsement,[4] the AHRQ QIs annually undergo ICD-10-CM/PCS code reviews, fiscal year coding updates, updates to the risk adjustment models, and reliability and validity testing.[5] Based on the literature review and empirical testing, the indicators are refined over time.

We limited the analysis to QIs for which we had the power to detect a 15% change in the mean during weeks with high COVID-19. This power analysis aimed to reduce the probability of Type 1 and Type 2 errors (i.e., false positive and false negative results). It was conducted before running regressions that measured changes in QIs.

Because the power analysis was conducted before running the main regressions, there are some differences from the principal analysis. First, it included patients with pneumonia and weeks 11-17, initially included in the analysis plan but excluded later to reduce the likelihood of including patients with unmeasured COVID-19 in the sample. Because of this difference in the sample, the means reported in eTable 2 differ from those reported in Table 5. For simplicity, the power analysis also assumed independent, identically distributed observations. We used a more conservative approach in the principal analysis (which clustered standard errors at the hospital level to account for correlations in patient outcomes within hospitals).

The IQI and PSIs that met the inclusion criteria are shown in eTable 2. Column 3 of eTable 2 reports the number of observations in 2020 at the discharge level during weeks with high COVID-19 admissions. Because our study measured the change in QIs relative to 2019, our null hypothesis was that the discharge-level mean during weeks with high COVID-19 admissions remained at 2019 levels. In column 4, we calculated the standard error of the mean during high COVID-19 weeks under the null hypothesis. We then multiplied the standard error by 1.96 to determine the change required to detect a statistically significant effect at the 95% confidence level (in column 5). Finally, we divided this change by the mean to determine which outcomes had enough observations to detect a 15% change in the mean.

A 15% cutoff was chosen because it focused the analyses on QIs with large enough sample sizes to detect clinically significant changes during the pandemic while also including QIs that were of particular interest, such as the Pressure Ulcer Rate (PSI 03) and Hip Fracture Mortality Rate (IQI 19). Previous literature suggests that the pressure ulcer rate is sensitive to nursing care, and it was the focus of patient safety initiatives during the pandemic.[6-10] Compared to other conditions, hip fracture is less likely a sequela of COVID-19; thus, this QI minimized the risk of confounding bias from COVID-19 infection rates.

The power analysis yielded 7 indicators from the broader non-surgical non-obstetric QIs. The selected QIs were the Patient Safety Indicator for Pressure Ulcer Rate (PSI 03) and in-hospital mortality for acute myocardial infarction (IQI 15), heart failure (IQI 16), acute stroke (IQI 17), gastrointestinal hemorrhage (IQI 18), hip fracture (IQI 19), and percutaneous coronary intervention (IQI 30). The numerators, identifying adverse events, and denominators, identifying the patients at risk for each selected QI, are presented in eTable 3. Detailed ICD-10-CM codes can be found on the AHRQ QI website.[1,2]

© 2024 Meille G et al. *JAMA Network Open.*

eTable 2. Power Analysis for QIs, with bolded indicators included in main analyses[a,b]

Quality Indicator	Total patient volume, 2020 weeks 11-48	Total patient volume during weeks with high COVID-19 admissions[c]	QI rate mean in 2019, weeks 1-48[d]	SE of QI rate during high COVID-19 admissions, assuming no change in mean[c]	1.96*SE	1.96* SE/mean
**IQI 15: AMI Mortality**	276,659	20,085	4.763	0.150	0.295	6.2%
**IQI 16: Heart Failure Mortality**	554,957	40,962	2.364	0.075	0.147	6.2%
**IQI 17: Acute Stroke Mortality**	336,036	23,794	6.490	0.160	0.313	4.8%
**IQI 18: GI Hemorrhage Mortality**	275,084	20,691	2.216	0.102	0.201	9.1%
**IQI 19: Hip Fracture Mortality**	149,756	11,869	1.804	0.122	0.239	13.3%
**IQI 30: PCI Mortality**	222,345	15,146	3.072	0.140	0.275	8.9%
PSI 02: Death in Low-Mortality DRGs	924,729	59,943	0.044	0.009	0.017	38.2%
**PSI 03: Pressure Ulcer**	7,630,346	512,599	0.0572	0.0033	0.0065	11.4%
PSI 06: Iatrogenic Pneumothorax	10,987,418	763,565	0.016	0.001	0.003	17.7%
PSI 07: CVC-RBSI	9,302,970	653,381	0.009	0.001	0.002	25.8%
PSI 08: In Hospital Fall with Hip Fracture	11,659,755	799,660	0.007	0.001	0.002	26.1%
PSI 15: Abdominopelvic APL	2,263,446	148,964	0.096	0.008	0.016	16.4%

Abbreviations: AMI, acute myocardial infarction; APL, accidental puncture or laceration; CVC-RBSI, central venous catheter-related blood stream infection; DRG, diagnosis-related group; GI gastrointestinal; IQI, Inpatient Quality Indicator; PCI, percutaneous coronary intervention; PSI, Patient Safety Indicator; SE, standard error.
[a]Source: Agency for Healthcare Research and Quality (AHRQ), Healthcare Cost and Utilization Project (HCUP) State Inpatient Databases (SID), 36 states, 2019-2020.
[b]Includes all patients without COVID-19. IQI 20 (pneumonia mortality), surgical, and delivery-related metrics not shown. Indicators included in main analysis (bolded) had sufficient power to detect a 15% change in mean at the 5% significance level (final column).
[c]Hospital-weeks categorized as high COVID-19 admissions if COVID-19 admissions per 100 beds were ≥15.
[d]Pressure ulcer means reported per 1,000 admissions, while all other means reported per 100 admissions.

© 2024 Meille G et al. *JAMA Network Open.*

eTable 3. Description of Selected Quality Indicators

Selected Quality Indicator	Description
PSI 03: Pressure Ulcer Rate	Stage 3 or 4 (or unstageable) pressure ulcers (secondary diagnosis not present on admission) per 1,000 hospital discharges of surgical or medical patients ages 18 years and older. Excludes discharges with length-of-stay less than 3 days; with a principal diagnosis of stage 3 or 4 (or unstageable) pressure ulcer or deep tissue injury at the same anatomic site; with severe burns; or with exfoliative skin disorders; and obstetric discharges. Excludes numerator events with a secondary diagnosis code for deep tissue injury or unstageable pressure ulcer present on admission at the same anatomic site.[2]
IQI 15: AMI Mortality	In-hospital deaths per 100 hospital discharges with a principal diagnosis of AMI for patients ages 18 years and older. Excludes transfers to another hospital, discharges admitted from a hospice facility, and obstetric discharges.[1]
IQI 16: Heart Failure Mortality	In-hospital deaths per 100 hospital discharges with a principal diagnosis of heart failure for patients ages 18 years and older. Excludes discharges with a procedure for heart transplant, discharges admitted from a hospice facility, transfers to another hospital, and obstetric discharges.[1]
IQI 17: Acute Stroke Mortality	In-hospital deaths per 100 hospital discharges with a principal diagnosis of acute stroke for patients ages 18 years and older. Includes metrics for discharges grouped by type of stroke. Excludes transfers to another hospital, discharges admitted from a hospice facility, and obstetric discharges.[1]
IQI 18: GI Hemorrhage Mortality	In-hospital deaths per 100 discharges with a principal diagnosis of gastrointestinal hemorrhage; or a secondary diagnosis of esophageal varices with bleeding along with a qualifying underlying principal diagnosis, for patients ages 18 years and older. Excludes discharges with a procedure for liver transplant, discharges admitted from a hospice facility, transfers to another hospital, and obstetric discharges.[1]
IQI 19: Hip Fracture Mortality	In-hospital deaths per 100 hospital discharges with hip fracture as a principal diagnosis for patients ages 65 years and older. Excludes periprosthetic fracture discharges, discharges admitted from a hospice facility, transfers to another hospital, and obstetric discharges.[1]
IQI 30: PCI Mortality	In-hospital deaths per 100 discharges with a procedure for percutaneous coronary intervention (PCI), for patients ages 40 years and older. Excludes transfers to another hospital, and obstetric discharges.[1]

Abbreviations: AMI, acute myocardial infarction; GI, gastrointestinal; IQI, Inpatient Quality Indicator; PCI, percutaneous coronary intervention; PSI, Patient Safety Indicator;

© 2024 Meille G et al. *JAMA Network Open.*

eAppendix 2. Background and Additional Results for Elixhauser Comorbidity Index Refined

The Elixhauser Comorbidity Software Refined for ICD-10-CM (hereafter Comorbidity Index), developed by AHRQ, creates a weighted index of 38 patient comorbidities. These comorbidities specify pre-existing conditions among non-maternal adult patients 18 years and older, based on secondary diagnoses (i.e., comorbidities or conditions that co-exist at the time of admission, that develop subsequently or affect the treatment received or length of stay) listed on hospital administrative records.[11] Developed initially using ICD-9-CM-coded data from the Healthcare Cost and Utilization Project (HCUP) from one state[12] and replicated with 18 states,[13] the Comorbidity Index was re-evaluated and updated by clinical experts using HCUP data from 45 States to identify 38 comorbidities (eTable 4), some of which are required to be present on admission if the condition cannot be assumed to be pre-existing at the time of the hospital stay.

The mortality-weighted Comorbidity Index was developed using 2018 HCUP State Inpatient Databases (SID) from 44 states and the District of Columbia to predict in-hospital mortality based on the 38 comorbidity measures. [14,15] One hundred bootstrapped replications (each based on a 5 percent random sample of the data) of backward stepwise logistic regression models were estimated with in-hospital mortality as the outcome variable. Two comorbidities were excluded because they had a very low observed in-hospital mortality rate and were highly correlated with another comorbidity measure (i.e., diabetes without chronic complications and uncomplicated hypertension). Three comorbidities (autoimmune disorders, solid tumor without metastasis, in situ; peptic ulcer with bleeding; and valvular disease) that were retained in less than 20 percent of the replications were also excluded from the final logistic model. The final logistic regression model for in-hospital mortality included 33 comorbidity measures (c-statistic of 0.777 for v2022.1 using 2018 data). Index weights were generated for each comorbidity to create a single index that accounts for interactions between comorbidities.

A similar approach was used to derive an index for 30-day readmissions. It was developed using the HCUP Nationwide Readmissions Database (NRD), 2018, limited to 26 states and without using the discharge weights. The model predicted 30-day readmissions based on the 38 comorbidity measures. Of the 38 comorbidities, 30 comorbidity measures were retained in the final model for readmissions (c-statistic of 0.634 for v2022.1 using 2018 data).

© 2024 Meille G et al. *JAMA Network Open.*

eTable 4. List of Comorbidities in Elixhauser Comorbidity Software Refined, v2022.1, Observed In-Hospital Mortality Rates and Index Weights, Observed 30-Day All-Cause Readmission Rates and Index Weights.[16]

Comorbidity Measure	Observed In-Hospital Mortality Rate (per 100 discharges)[a]	Risk of In-Hospital Mortality Index Weight[c]	Observed 30-Day All Cause Readmission Rate (per 100 events)[b]	Risk of 30-Day All-Cause Readmission Index Weight[c]
AIDS	2.15	-4	18.53	5
Alcohol abuse	2.59	-1	15.88	3
Anemias due to other nutritional deficiencies	3.53	-3	19.25	5
Autoimmune disorders	2.57	-1	14.93	2
Chronic blood loss (iron deficiency)	2.95	-4	16.86	2
Cancer – leukemia	5.63	9	21.76	10
Cancer – lymphoma	4.78	6	19.48	7
Cancer – metastatic	8.81	23	21.06	11
Cancer – solid tumor w/o metastasis, in situ	1.63	0	12.07	0
Cancer – solid tumor w/o metastasis, malignant	5.09	10	18.72	7
Cerebrovascular disorder	4.99	5	15.39	0
Heart failure	5.15	15	19.81	7
Coagulopathy	7.74	15	19.66	3
Dementia	4.84	5	14.94	1
Depression	1.80	-9	14.63	2
Diabetes w/ chronic complications	3.19	-2	18.12	4
Diabetes, w/o chronic complications	2.23	0	12.57	0
Drug abuse	1.45	-7	16.90	6
Hypertension, complicated	4.27	1	18.03	0
Hypertension, uncomplicated	1.75	0	10.77	0
Liver disease, mild	2.96	2	16.72	3
Liver disease and failure, moderate to severe	8.83	17	24.95	10
Chronic pulmonary disease	3.23	2	16.48	4
Neurological disorders affecting movement	3.23	-1	14.86	1
Other neurological disorder	2.78	23	16.69	2
Seizures and epilepsy	9.34	2	17.70	5
Obesity	3.74	-7	12.46	-2
Paralysis	1.80	4	15.70	3
Perivascular disorders	4.60	3	15.91	1
Psychoses	4.00	-9	17.24	6
Pulmonary circulatory disorders	1.55	4	20.08	3
Renal (kidney) failure and disease, moderate	5.07	3	17.55	4
Renal (kidney) failure and disease, severe	3.85	8	23.61	8
Hypothyroidism	4.90	-3	14.04	0
Other thyroid disorders	2.60	-8	12.74	0

© 2024 Meille G et al. *JAMA Network Open.*

eTable 4. (continued) List of Comorbidities in Elixhauser Comorbidity Software Refined, v2022.1, Observed In-Hospital Mortality Rates and Index Weights, Observed 30-Day All-Cause Readmission Rates and Index Weights.[16]

Comorbidity Measure	Observed In-Hospital Mortality Rate (per 100 discharges)[a]	Risk of In-Hospital Mortality Index Weight[c]	Observed 30-Day All Cause Readmission Rate (per 100 events)[b]	Risk of 30-Day All-Cause Readmission Index Weight[c]
Peptic ulcer with bleeding	1.82	0	16.62	2
Valvular disease	3.47	0	16.53	0
Weight loss	7.36	14	20.12	6

Abbreviations: AIDS, acquired immune deficiency syndrome, w/, with; w/o, without
[a]Source: Agency for Healthcare Research and Quality (AHRQ), Healthcare Cost and Utilization Project (HCUP), State Inpatient Databases (SID), 2018, 44 States and the District of Columbia.
[b]Source: Agency for Healthcare Research and Quality (AHRQ), Healthcare Cost and Utilization Project (HCUP), Nationwide Readmissions Database (NRD), 2018, limited to 26 States and without the use of the discharge weights included in the NRD.
[c]The index weight for each comorbidity was calculated as the value of its regression coefficient divided by the absolute value of the regression coefficient for the comorbidity with the smallest absolute value, rounded to the nearest integer. A negative comorbidity weights indicates a protective relationship with in-hospital mortality (or readmission) in the model.

© 2024 Meille G et al. *JAMA Network Open.*

eTable 5. Elixhauser Comorbidity Index Refined for Readmission Among At-Risk Patients for Selected Quality Indicators in 2019 and Change in 2020 During Weeks of Low and High COVID-19 Admissions[a]

Quality Indicator (N=Number of Hospital-Weeks)	Mean Elixhauser Comorbidity Index Refined for 30-day Readmission among At-Risk Patients at Hospital-Week Level			
	Mean in 2019 (SE)[b,c]	Change during Low COVID-19 Weeks [b,c,d]	Change during High COVID-19 Weeks [b,c,d]	Difference in the Change During High vs. Low COVID-19 Weeks [b,c,d]
		Estimate (95% confidence interval)		
PSI 03: Pressure Ulcer (N=235,268)	9.61 (0.04)	0.56 (0.51 to 0.61)	0.28 (0.22 to 0.34)	-0.28 (-0.36 to -0.20)
Average of Mortality Indicators Below[d] (N=667,592)	11.16 (0.05)	0.36 (0.28 to 0.44)	0.03 (-0.06 to 0.12)	-0.33 (-0.45 to -0.22)
IQI 15: AMI Mortality (N=102,014)	7.88 (0.05)	-0.05 (-0.21 to 0.12)	-0.27 (-0.47 to -0.07)	-0.22 (-0.48 to 0.04)
IQI 16: Heart Failure Mortality (N=149,790)	17.22 (0.05)	0.38 (0.27 to 0.50)	-0.02 (-0.16 to 0.11)	-0.41 (-0.58 to -0.24)
IQI 17: Acute Stroke Mortality (N=111,338)	8.17 (0.04)	0.35 (0.21 to 0.49)	0.11 (-0.05 to 0.26)	-0.25 (-0.46 to -0.04)
IQI 18: GI Hemorrhage Mortality (N=119,286)	11.14 (0.05)	0.68 (0.51 to 0.85)	0.38 (0.18 to 0.57)	-0.30 (-0.57 to -0.04)
IQI 19: Hip Fracture Mortality (N=93,292)	7.32 (0.04)	0.47 (0.28 to 0.66)	-0.03 (-0.26 to 0.21)	-0.49 (-0.79 to -0.19)
IQI 30: PCI Mortality (N=91,872)	6.81 (0.05)	0.38 (0.21 to 0.55)	0.03 (-0.18 to 0.23)	-0.35 (-0.62 to -0.09)

Abbreviations: AMI, acute myocardial infarction; GI, gastrointestinal; IQI, Inpatient Quality Indicator; PCI, percutaneous coronary intervention; PSI, Patient Safety Indicator.
[a]Source: Agency for Healthcare Research and Quality (AHRQ), Healthcare Cost and Utilization Project (HCUP) State Inpatient Databases (SID), 36 states, 2019-2020.
[b]Means, regressions, and average of mortality indicators were weighted by the number of admissions for each QI in each hospital-week.
[c]Standard errors were clustered at the hospital level.

© 2024 Meille G et al. *JAMA Network Open.*

[d]Changes were reported for weeks 18-48 based on a hospital-week level linear regression of 2019 and 2020 data. Fixed effects for hospital and weeks were included in each regression.

© 2024 Meille G et al. *JAMA Network Open.*

eTable 6. Sample Characteristics[a]

Characteristic	Sample Mean (SD)							
	All (N= 19 111 629)	PSI 03: Pressure Ulcer (N = 17 779 389)	IQI 15: AMI Mortality (N = 706 786)	IQI 16: Heart Failure Morality (N = 1 357 409)	IQI 17: Acute Stroke Mortality (N = 791 544)	IQI 18: GI Hemor-rhage Mortality (N = 679 099)	IQI 19: Hip Fracture Mortality (N = 350 859)	IQI 30: PCI Mortality (N = 559 506)
Age in years	63.0 (18.0)	62.7 (18.2)	66.4 (13.6)	70.6 (14.8)	69.3 (14.5)	67.5 (16.4)	81.9 (8.5)	66.1 (11.8)
Female, %	50.3 (50.0)	50.9 (50.0)	36.9 (48.3)	47.1 (49.9)	49.3 (50.0)	47.2 (49.9)	70.7 (45.5)	32.7 (46.9)
Hispanic, %	9.9 (29.9)	9.9 (29.9)	9.2 (29.0)	8.7 (28.2)	9.3 (29.0)	10.5 (30.6)	5.8 (23.3)	8.4 (27.8)
Non-Hispanic Black, %	16.6 (37.2)	16.7 (37.3)	11.9 (32.4)	23.6 (42.5)	18.1 (38.5)	16.6 (37.2)	4.1 (19.7)	9.7 (29.6)
Non-Hispanic White, %	66.4 (47.2)	66.3 (47.3)	70.3 (45.7)	61.5 (48.7)	64.0 (48.0)	65.3 (47.6)	84.4 (36.3)	73.4 (44.2)
Other race or ethnicity, %	5.3 (22.4)	5.3 (22.3)	6.2 (24.0)	4.7 (21.2)	6.5 (24.7)	6.0 (23.7)	4.1 (19.9)	6.0 (23.7)
Missing race ethnicity, %	1.8 (13.4)	1.8 (13.4)	2.4 (15.4)	1.5 (12.2)	2.1 (14.3)	1.7 (12.9)	1.6 (12.6)	2.5 (15.5)
Comorbidity Index	7.4 (15.1)	7.3 (15.3)	6.3 (12.6)	16.3 (11.7)	11.9 (15.6)	9.7 (15.5)	6.3 (12.3)	4.7 (11.4)
Medicare[b], %	55.7 (49.7)	55.4 (49.7)	54.6 (49.8)	70.2 (45.7)	62.0 (48.5)	62.9 (48.3)	90.6 (29.2)	54.5 (49.8)
Medicaid[b], %	15.1 (35.8)	15.4 (36.1)	9.6 (29.4)	12.1 (32.7)	9.9 (29.8)	11.8 (32.3)	1.0 (9.9)	8.6 (28.1)
Private insurance[b], %	21.0 (40.7)	20.9 (40.7)	26.3 (44.0)	11.8 (32.2)	20.1 (40.0)	17.7 (38.2)	6.1 (23.9)	27.7 (44.8)
Self-pay[b], %	5.1 (22.0)	5.0 (21.9)	6.1 (24.0)	3.7 (18.9)	5.3 (22.5)	5.0 (21.8)	0.6 (7.6)	5.7 (23.1)
Other payers[b], %	3.0 (17.2)	3.1 (17.2)	3.2 (17.6)	2.0 (14.2)	2.6 (16.0)	2.4 (15.4)	1.7 (12.8)	3.3 (18.0)
No. of procedures	1.9 (2.7)	1.9 (2.7)	4.0 (3.1)	1.1 (2.0)	1.2 (2.3)	2.3 (2.1)	1.5 (1.1)	4.8 (2.5)
In-hospital mortality, %	1.7 (13.0)	1.6 (12.4)	4.0 (19.5)	2.0 (13.9)	5.3 (22.4)	1.9 (13.6)	1.4 (11.6)	2.4 (15.4)

Abbreviations: AMI, acute myocardial infarction; GI, gastrointestinal; IQI, Inpatient Quality Indicator; PCI, percutaneous coronary intervention; PSI, Patient Safety Indicator; Comorbidity Index, Elixhauser Comorbidity Refined Index.

[a]Source: Agency for Healthcare Research and Quality (AHRQ), Healthcare Cost and Utilization Project (HCUP) State Inpatient Databases (SID), 36 states, 2019-2020.

[b]Insurance type reflects expected primary payer. Other payers includes: Federal and local government programs (e.g., TRICARE, Indian Health Service, Black Lung, Title V) and Worker's Compensation.

© 2024 Meille G et al. *JAMA Network Open.*

eTable 7. Hospital Quality Indicators in 2019 and Adjusted Changes in 2020 by Level of COVID-19 Admissions[a]

Quality Indicator (N=Number of Discharges)	Mean in 2019 (SE)[b]	Change in during 2020, by COVID-19 Admission Rate per 100 beds, (95% confidence interval in parentheses)[b,c]				
		Rate <1	Rate 1-4.9	Rate 5-9.9	Rate 10-14.9	Rate ≥15
PSI 03: Pressure Ulcer (N=17,779,128)	0.37 (0.02)	-0.12 (-0.18 to -0.06)	-0.07 (-0.11 to -0.04)	-0.06 (-0.10 to -0.02)	-0.05 (-0.10 to 0.00)	-0.03 (-0.10 to 0.03)
Average of Mortality Indicators Below[d] (N=4,429,736)	2.83 (0.03)	-0.08 (-0.17 to 0.02)	0.01 (-0.04 to 0.07)	0.04 (-0.03 to 0.11)	0.09 (-0.01 to 0.19)	0.22 (0.1 to 0.34)
IQI 15: AMI Mortality (N=703,457)	3.87 (0.04)	0.02 (-0.16 to 0.20)	0.10 (-0.00 to 0.21)	0.13 (-0.01 to 0.27)	0.25 (0.05 to 0.45)	0.21 (-0.04 to 0.45)
IQI 16: Heart Failure Mortality (N=1,354,741)	1.90 (0.03)	0.02 (-0.13 to 0.16)	0.12 (0.04 to 0.20)	0.23 (0.13 to 0.33)	0.21 (0.06 to 0.36)	0.42 (0.24 to 0.59)
IQI 17: Acute Stroke Mortality (N=787,982)	5.36 (0.10)	-0.54 (-0.86 to -0.22)	-0.36 (-0.54 to -0.18)	-0.51 (-0.73 to -0.30)	-0.34 (-0.65 to -0.04)	-0.17 (-0.50 to 0.17)
IQI 18: GI Hemorrhage Mortality (N=675,849)	1.78 (0.03)	0.09 (-0.12 to 0.29)	0.13 (0.02 to 0.24)	0.11 (-0.03 to 0.25)	0.06 (-0.16 to 0.29)	0.29 (0.06 to 0.52)
IQI 19: Hip Fracture Mortality (N=348,496)	1.36 (0.03)	-0.07 (-0.30 to 0.16)	-0.09 (-0.23 to 0.04)	0.06 (-0.11 to 0.22)	0.15 (-0.11 to 0.41)	0.33 (0.05 to 0.61)
IQI 30: PCI Mortality (N=559,211)	2.41 (0.04)	0.03 (-0.21 to 0.27)	0.10 (-0.03 to 0.23)	0.14 (-0.04 to 0.32)	0.21 (-0.06 to 0.48)	0.14 (-0.19 to 0.46)

Abbreviations: AMI, acute myocardial infarction; GI gastrointestinal; PCI, percutaneous coronary intervention; PSI, Patient Safety Indicator; IQI, Inpatient Quality Indicator

[a]Source: Agency for Healthcare Research and Quality (AHRQ), Healthcare Cost and Utilization Project (HCUP) State Inpatient Databases (SID), 36 states, 2019-2020.

[b]Standard errors clustered at the hospital level. Pressure ulcer rates were examined per 1,000 admissions and mortality was examined per 100 admissions.

[c]Changes were reported for weeks 18-48 based on a discharge-level linear regression of 2019 and 2020 data. Each regression controlled for sex, an age spline, interactions between sex and the age spline, Medicare Severity-Diagnosis Related Groups (MS-DRG) (or Major Diagnostic Category if <1,000 observations for a MS-DRG), and 38 comorbidities. Fixed effects for hospital-months were included in each regression.

[d]Average was weighted by number of discharges for each mortality quality indicator.

© 2024 Meille G et al. *JAMA Network Open.*

eReferences

1. Agency for Healthcare Research and Quality. Technical Specifications For Inpatient Quality Indicators. Accessed November 30, 2023. https://qualityindicators.ahrq.gov/Archive/iqi_techspec/icd10_v2022
2. Agency for Healthcare Research and Quality. Technical Specifications For Patient Safety Indicators. Accessed November 30, 2023. https://qualityindicators.ahrq.gov/Archive/psi_techspec/icd10_v2022
3. Agency for Healthcare Research and Quality. Toolkit for Using the AHRQ Quality Indicators. Updated March 2017. https://www.ahrq.gov/patient-safety/settings/hospital/resource/qitool/index.html
4. Agency for Healthcare Research and Quality. *Rationale for not seeking NQF Endorsement for Quality Indicators*. 2021. https://qualityindicators.ahrq.gov/Downloads/News/AHRQ_Rationale4notseekingNQFendorsement-May2021.pdf
5. Agency for Healthcare Research and Quality. *Quality Indicator Empirical Methods, v2023*. 2023. https://qualityindicators.ahrq.gov/Downloads/Resources/Publications/2023/Empirical_Methods_2023.pdf
6. Blume KS, Dietermann K, Kirchner-Heklau U, et al. Staffing levels and nursing-sensitive patient outcomes: Umbrella review and qualitative study. *Health Serv Res*. Oct 2021;56(5):885-907. doi:10.1111/1475-6773.13647
7. Engineer LD, Winters BD, Weston CM, et al. Hospital Characteristics and the Agency for Healthcare Research and Quality Inpatient Quality Indicators: A Systematic Review. *J Healthc Qual*. Sep-Oct 2016;38(5):304-13. doi:10.1097/JHQ.0000000000000015
8. Savitz LA, Jones CB, Bernard S. Quality Indicators Sensitive to Nurse Staffing in Acute Care Settings. In: Henriksen K, Battles JB, Marks ES, Lewin DI, eds. *Advances in Patient Safety: From Research to Implementation (Volume 4: Programs, Tools, and Products)*. 2005. *Advances in Patient Safety*.
9. Polancich S, Hall AG, Miltner R, et al. Learning During Crisis: The Impact of COVID-19 on Hospital-Acquired Pressure Injury Incidence. *J Healthc Qual*. May-Jun 01 2021;43(3):137-144. doi:10.1097/JHQ.0000000000000301
10. Team V, Jones A, Weller CD. Prevention of Hospital-Acquired Pressure Injury in COVID-19 Patients in the Prone Position. *Intensive Crit Care Nurs*. Feb 2022;68:103142. doi:10.1016/j.iccn.2021.103142
11. Centers for Medicare & Medicaid Services. *ICD-10-CM Official Guidelines for Coding and Reporting FY 2019 (October 1, 2018 - September 30, 2019)*. Accessed December 6, 2023. https://www.cms.gov/Medicare/Coding/ICD10/Downloads/2019-ICD10-Coding-Guidelines-.pdf
12. Elixhauser A, Steiner C, Harris DR, Coffey RM. Comorbidity measures for use with administrative data. *Med Care*. Jan 1998;36(1):8-27. doi:10.1097/00005650-199801000-00004
13. Moore BJ, White S, Washington R, Coenen N, Elixhauser A. Identifying Increased Risk of Readmission and In-hospital Mortality Using Hospital Administrative Data: The AHRQ Elixhauser Comorbidity Index. *Med Care*. Jul 2017;55(7):698-705. doi:10.1097/MLR.0000000000000735
14. Sharma N, Schwendimann R, Endrich O, Ausserhofer D, Simon M. Comparing Charlson and Elixhauser comorbidity indices with different weightings to predict in-hospital mortality: an analysis of national inpatient data. *BMC Health Serv Res*. Jan 6 2021;21(1):13. doi:10.1186/s12913-020-05999-5
15. Sharabiani MT, Aylin P, Bottle A. Systematic review of comorbidity indices for administrative data. *Med Care*. Dec 2012;50(12):1109-18. doi:10.1097/MLR.0b013e31825f64d0
16. Agency for Healthcare Research and Quality. *User Guide: Elixhauser Comorbidity Software Refined for ICD-10-CM Diagnoses v2023.1*. 2022. https://hcup-us.ahrq.gov/toolssoftware/comorbidityicd10/CMR-User-Guide-v2023-1.pdf

© 2024 Meille G et al. *JAMA Network Open.*
