# Supplementary material for: COVID-19 Admission Rates and Changes in Care Quality in US Hospitals
Source: JAMA Netw Open. 2024 May 24;7(5):e2413127. doi: 10.1001/jamanetworkopen.2024.13127 (PMC11127115; doi:10.1001/jamanetworkopen.2024.13127)
Supplement: Supplement 2. — Data Sharing Statement [file jamanetwopen-e2413127-s002.pdf]

Data Sharing Statement

Meille. COVID-19 Admission Rates and Changes in Care Quality in US Hospitals. *JAMA Netw Open*. Published May 24, 2024. doi:10.1001/jamanetworkopen.2024.13127

Data
Data available: No

Additional Information
Explanation for why data not available: This study used restricted data from the Healthcare Cost and Utilization Project (HCUP). It contained exact admission dates, which cannot be publicly shared because of restrictions in the data use agreement between the Agency for Healthcare Research and Quality and HCUP partner states.
